# A Novel Mutation of *Hyaluronan Synthase 2* Gene in Chinese Children with Ventricular Septal Defect

**DOI:** 10.1371/journal.pone.0087437

**Published:** 2014-02-18

**Authors:** Xiaomei Zhu, Xiaopeng Deng, Guangying Huang, Jing Wang, Jingwen Yang, Si Chen, Xu Ma, Binbin Wang

**Affiliations:** 1 Graduate School of Peking Union Medical College, Beijing, China; 2 National Research Institute for Family Planning, Beijing, China; 3 Department of Obstetrics and Gynecology, Shengjing Hospital of China Medical University, Shenyang, China; 4 World Health Organization Collaborating Centre for Research in Human Reproduction, Beijing, China; 5 Children's Hospital of Fudan University, Shanghai, China; 6 Department of Medical Genetics, School of Basic Medical Sciences, Capital Medical University, Beijing, China; Temple University, United States of America

## Abstract

As a major product of extracellular matrix (ECM), Hyaluronic acid (HA) is involved in early cardiac development and mainly synthesized by Hyaluronan synthase 2 (HAS2) during embryogenesis. Targeted deletion of HAS2 gene in mice led to obvious cardiac and vascular defects. To clarify the potential association of the mutation in HAS2 with the development of congenital heart disease (CHD), in this study, we sequenced the coding region of HAS2 and identified a novel non-synonymous variant c.A1496T (p.Glu499Val) in one of 100 non-syndromic Ventricular Septal Defect (VSD) patients. The variant was not observed in 250 controls. In addition, to determine the contribution of HAS2 variant in VSD, we compared HA content in supernatant using HA quantitative analysis and found that the mutation obviously affected the HA synthetic activity of HAS2. To our knowledge, this is the first time that the mutation in HAS2 was found in Chinese VSD patients, which suggested that HAS2 may be involved in the etiology of non-syndromic VSD and have the vital function in the development of heart septum.

## Introduction

CHD is now recognized as the leading non-infectious cause of birth defects related to inadequate function of heart for an estimated incidence of 5% of all live births [1]. The formation process of heart is complex and integrative, including a variety of molecular mechanisms and morphogenetic events [2]. Subtle disturbance at any point during cardiac development leads to a large spectrum of CHD. To our knowledge, many genetic factors have been found to be involved in the complex biological processes of heart development, such as transcription factors [3]–[5], MicroRNAs [6], [7] and ECM products [8]–[10]. VSD is the most frequent form of CHD worldwide [11], so it is important to understand the mechanisms of septa and valve development.

As a major component of ECM, it is widely recognized that HA highly expresses in the developing heart and has a prominent role in cell migration and transformation, especially in endocardial epithelial-mesenchymal transformation (EMT) during the early development of cardiovascular system [12]–[14]. Though HA can be produced in vivo by three hyaluronan synthase isoenzymes (HAS) [15]–[18], most of HA is synthesized by HAS2 during embryonic development [19]. The human HAS2 gene locates on 8q24.12, and encodes a 552 amino acid protein, with a predicted structure that includes a catalytic region of the enzyme in the central domain and clusters of 7 transmembrane domains [16], [17], [20], [21]. Targeted deletion of the HAS2 gene in mice exhibits obvious cardiac and vascular defects [19]. Although HAS2 has been shown to affect the formation of endocardial cushions and the process of EMT during the heart development, there is no report about the relationship between HAS2 and CHD in human.

From the discussion above, we hypothesize that HAS2 may contribute to the development of CHD. The primary aim of the present work was to carry out mutational screening of the HAS2 gene in Chinese VSD children. Furthermore, we showed the influence of mutation on the catalytic activity of HAS2 and provided insight into the potential etiology of VSD.

## Materials and Methods

### Study Population

In this study, 100 non-syndromic VSD patients from Chinese Han population and 250 ethnically matched unrelated individual normal controls with no reported cardiac phenotype were recruited from Lanzhou University. Written informed consent was signed by participants or their guardians. The study conformed to the ethical guidelines of the 1975 Declaration of Helsinkiwas and approved by the Ethics Committee of the National Research Institute for Family Planning.

All participants underwent an extensive, standardized examination, which included anthropometric measurement, physical examination for dysmorphism and malformation, radiological evaluation. The patients also underwent a chest X-ray examination, electrocardiogram, and ultrasonic echocardiogram.

### DNA analysis and bioinformatics analysis

Genomic DNA was extracted from peripheral blood leukocytes using the QIAamp RNA Blood Mini Kit (Qiagen, Valencia, CA). The human HAS2 gene is located on 8q24.12 including three exons. Three pairs of HAS2 gene-specific primers (shown in Table 1) to amplify coding region of HAS2 were designed by Primer 5.0 software. PCR products were sequenced using the appropriate PCR primers and the BigDye Terminator Cycle Sequencing kit (Applied Biosystems, Foster City, CA, USA) and run on an automated sequence, ABI 3730XL (Applied Biosystems) to perform mutational analysis.

**Table 1:** HAS2 gene sequencing primers for various exons.

Exon	Primer ID	Sequence (5′ to 3′)	Size(bp)
1	HAS2-1F	TGGGCGAGAAATTGAGTGTT	775
	HAS2-1R	GGTCTCCACATTCCTGCCA	
2	HAS2-2F	CAGAGGGCCAGATGAACACT	986
	HAS2-2R	GGATCTGCTTCACTGCCTCT	
3	HAS2-3F	TCACCATCAAAGAATCGCAAC	1244
	HAS2-3R	ATCAGATAATGCCACCAAAGGA	

The novel variant found in sequencing was first determined in the NHLBI Exome Sequencing Project (ESP) Exome Variant Server, EvoSNP-DB, the National Centre for Biotechnology Information (NCBI) human SNP database (dbSNP) and the 1000 Genome Project database (http://browser.1000genomes.org/). The conservation of HAS2 gene was analyzed by CLC Main Workbench Software (Aarhus, Denmark).

Polyphen and Sift were used to predict the effect of non-synonymous substitution on HAS2 function.

### Site-directed mutagenesis and plasmid construction

The open reading frame (ORF) of wild-type HAS2 was amplified by PCR from cDNA obtained from OriGene TrueClone. Site-directed mutagenesis was constructed using the QuikChange Lightning site-directed mutagenesis kit (Stratagene, La Jolla, CA, USA) with appropriate primers designed by siDESIGN Center (Thermo scientific, http://www.dharmacon.com/designcenter/DesignCenterPage.aspx) to generate human HAS2's ORF bearing c.A1496T (p.Glu499Val). The ORFs of wild-type or mutant HAS2 was ligated into the pcDNA3.1(+) vector which was dual digested by BamHI and XbaI and pEGFP-N1 vector which was dual digested by BamHI and HindIII. All the clones were confirmed by sequencing. The nucleotide sequences of the PCR primers used above are shown in Table 2.

**Table 2:** Primers used in plasmid construction.

Primer	Sequence
Primers used to construct pcDNA3.1-HAS2 (5′ to 3′)	Forward: CGGGATCCGCCACCATGCATTGTGAGAGGTTTCTATG
	Reverse: GCTCTAGATCATACATCAAGCACCATGTCAT
Primers used to construct pcDNA3.1-HAS2 E499V mutant (5′ to 3′)	Forward: TGTGATTTTCACCATTTATAAGGTGTCTAAAAGGCCATTTTCAGAAT
	Reverse: ACACTAAAAGTGGTAAATATTCCACAGATTTTCCGGTAAAAGTCTTA
Primers used to construct pEGFP-HAS2 (5′ to 3′)	Forward: CCCAAGCTTGCCACCATGCATTGTGAGAGGTTTCTATG
	Reverse: CGGGATCCCC TACATCAAGC ACCATGTC

### Cell culture and transient transfection

293T cells were cultured in Dulbecco's Modified Eagle Medium supplemented with 10% fetal bovine serum, 100 mg/ml penicillin, and 100 mg/ml streptomycin in a humanized atmosphere containing 5% CO_2_ at 37°C. All transfections were performed with Lipofectamine 2000 (Invitrogen Corporation, Carlsbad, CA, USA) according to the manufacturer's protocol.

### Subcellular localization

293T cells were plated in 6-well culture plates 24 h prior to transfection at approximately 70% confluency. GFP-HAS2 expression plasmid containing wild-type or mutant HAS2 was transfected using Lipofectamine 2000, according to the manufacturer's instructions. The empty vector pEGFP-N1 was transfected as a control. 30 h after transfection, the cells were analyzed by an inverted fluorescence microscope.

### HA Quantitative Assay

293T cells were plated at 2×10^6^cells/flask (25 cm^2^, Corning) at 24 h prior to transfection and transfected with equimolar amounts of pcDNA3.1-wild-type HAS2 or mutant HAS2. Culture supernatant was collected at 48 h, centrifugated, diluted 8 fold and analyzed for total amount of HA using HA kit (Tigsun Corporation, Beijing, China). HA quantitative assay procedures were operated according to the manufacturer's protocols. The values are normalized to total cell number expressed as µg/10^6^ cells. Cell was dissociated by trypsinization and the cell numbers were determined by hemacytometer count. Results are representative of 3 separate experiments. The significance of differences was calculated using the independent-samples t test.

## Results

### Genetic and bioinformatics analysis

**Figure 1:**
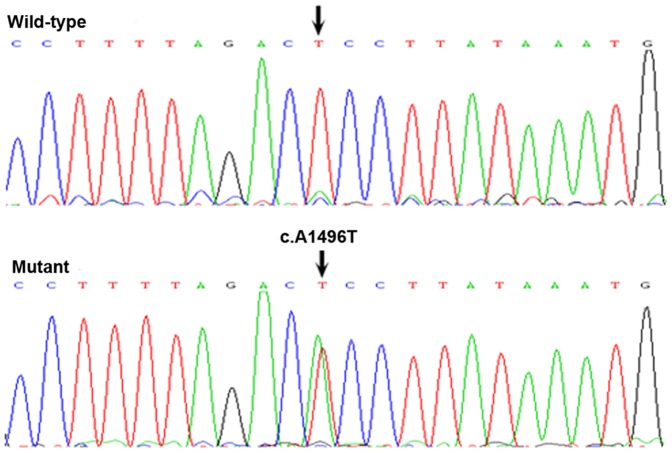
Mutation analysis of the HAS2 gene. The sequence chromatogram (reverse strand) shows a heterozygous T>A transition which resulted in the substitution of glutamic acid by valine at codon 499. The black arrows show the wild-type (the controls) and mutant point (patient).

**Figure 2:**
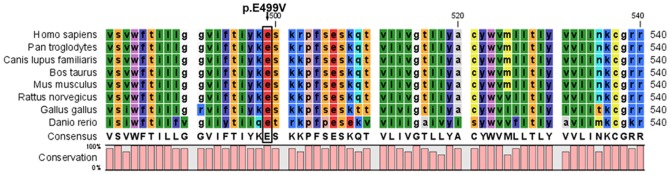
The alignment of the HAS2 sequence with the corresponding segments in different species was shown. It indicates that glutamic acid at position 499 is highly conserved. The black arrow indicates high conservation.

In the patient group, a novel non-synonymous substitution of c.A1496T (p.Glu499Val) was detected in a 6-year-old child (Fig. 1). Glu499 was highly conserved among many species (human, mice, rat, chimpanzee, dog, cow, chicken and zebrafish, shown in Fig. 2). The variant was not found in 250 controls and has not been listed in the NHLBI Exome Sequencing Project (ESP) Exome Variant Server, EvoSNP-DB, the NCBI dbSNP and the 1000 Genome Project database (http://browser.1000genomes.org/). Using two different algorithms (PolyPhen and Sift Functional analysis) to predict the effect of the mutation on HAS2 function, the novel non-synonymous substitution (p.Glu499Val) may be damaging.

### Subcellular localization

**Figure 3:**
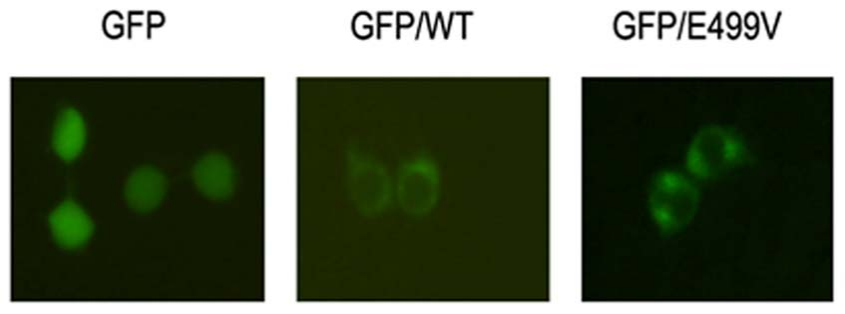
Effect of E499V mutation on subcellular localization of HAS2. 293T cells were transfected with empty vector pEGFP-N1, pEGFP-HAS2 wild-type, and the E499V mutant separately. After 30 h, transfected cells were then analyzed with an inverted fluorescence microscope to study the protein localization.

The effect of the mutation on the cellular localization of HAS2 was observed using subcellular localization. As shown in Fig. 3, GFP (control) was located in both the nucleus and cytoplasm, while both of wild-type and mutant HAS2 fusion proteins were mainly detected in the plasma membrane and cytoplasm of 293T cells. So the HAS2 mutation didn't alter its nuclear localization pattern.

### HA quantitative assay

**Figure 4:**
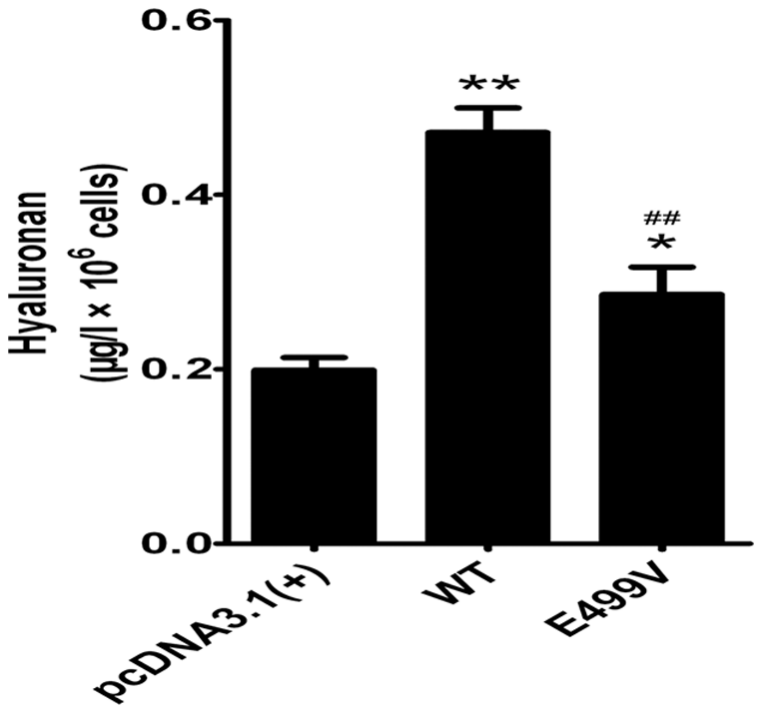
Effect of E499V mutation on HA synthesis. 293T cells were transfected with empty vector pcDNA3.1 (+), pcDNA3.1-HAS2 wild-type and the E499V mutant separately. HA content in media was determined as described in the methods section. Results are representative of three separate experiments. The significance of differences was calculated using the independent-samples t test. *p<0.05, **p<0.01 versus empty vector pcDNA3.1 (+); #p<0.05, ##p<0.01 versus wild-type.

HA quantitative assay was used to evaluate whether the mutation affected catalytic property of HAS2. We transfected the plasmid including wild-type or mutant HAS2 into 293T, and compared the production of HA. The wild-type HAS2 resulted in a 2.37-fold increase (t test, p<0.001) of HA production compared with control (pcDNA3.1 only). The mutant HAS2 resulted in an approximately 40% (t test, p<0.01) reduction of the catalytic activity compared with wild-type HAS2 (Fig. 4).

## Discussion

As the principal source of HA during the primary morphogenetic stage of embryo, HAS2 expression was induced by many signaling molecules involved in endocardial cushion formation to regulate HA content during embryonic cardiac development, including TBX2/Bmp2 [22], [23], MEKK [24], all-trans-retinoic acid [25] and β-catenin [26]. Disruption of HAS2 in mice results in death at embryonic day 9.5 and exhibits diverse abnormalities of the developing cardiovascular system, including lack of endocardial cushions and trabeculae [19], [27]. More importantly, the absent EMT in the atrioventricular (AV) canal from HAS2^−/−^ embryos can be rescued by HAS2 cDNA and the addition of purified HA [19]. During cardiac development, EMT results in formation of the endocardial cushions, which requires dynamic interaction between cells and ECM. Regarded as the primordia of membranous septa [28], endocardial cushions provide mesenchymal element for the septum [29]. Therefore, any interference in EMT or endocardial cushions can disturb the septal morphogenesis. Taken together, We propose that HAS2 may play an important role in septum development and be a good candidate gene for human VSD, but there is few evidence suggesting that HAS2 mutation are associated with VSD in human [10].

In present study, we screened the coding region sequence of the HAS2 gene in 100 Chinese VSD patients. As a result, one novel non-synonymous variant (p.Glu499Val) was discovered in a 6-year-old male patient and not identified in 250 controls. To our knowledge, this is the first HAS2 mutation found in VSD patients, suggesting that the alteration of HAS2 protein has a potential impact on the formation of ventricular septum.

In the previous studies, point-mutated and truncated HASs focusing on high conservative sites or regions showed reduced even quenched activity of wild-type HASs [30]–[33]. The mutation E499V which we screened from VSD patients was changed from a polarity negative glutamic acid into one aliphatic series amino acid valine. Furthermore, the mutation is highly conservative in many species (human, mice, rat, chimpanzee, dog, cow, chicken and zebrafish). Most importantly, the mutation was predicted by PolyPhen and Sift to have a potential effect on the function of HAS2. Therefore, this mutation should possibly have an adverse effect.

As one of the main constituents of ECM, HA can induce signal-transduction pathways and affect a variety of physiological cellular events, such as cell proliferation, differentiation and migration [34], [35] by interacting with cell-surface receptors (i.e., CD44, RHAMM, LYVE-1, HARE, TLR4) [36]–[41]. Remarkably, previous studies revealed that HA-activated ErbB2/3 receptors result in the activation of Wnt/ß-catenin and Ras signal pathways [27], [42] which are implicated in EMT during the formation of endocardial cushion and heart valves [43]. Meanwhile, it has been confirmed that the increased expression of HA is sufficient to induce EMT [44]. In addition, previous researchers have identified the mutations of genes involved in HA synthesis or associated pathways, such as UGDH [45], ERBB3 [46] and APC [6], [46] in the atrioventricular valve and septum defects patients. So HA is crucial for formation of the septal structures during embryonic development and any disturbance of HA may lead to septal defects.

In our study, the HA content was determined just in supernatant because most synthesized HA is secreted into cultured media [20]. Comparison of HA in the supernatants of transfected cells showed that the HA synthesis potency of mutant HAS2 displayed a decreased tendency compared with the wild-type. The result shows that the mutation we screened from VSD patients has an impact on the function of HAS2 and could have influence on the synthesis of HA, which strongly supports the speculation that the HA insufficiency and HAS2 mutation might affect the formation of septum.

Previous studies predicted that the catalytic regions of HASs were located on the central domains [15], but the mutation we found was located on C termini domain and had an obvious effect on HAS2 function. Up to now, the specific active domains of HAS2 have not yet been defined, so further experiment studies are necessary to clarify the functional significance of the non-synonymous variant of HAS2 in order to identify the role of HAS2 in VSD.

In conclusion, the mutational analysis of HAS2 in 100 Chinese VSD patients revealed one novel non-synonymous variant, c.A1496T (p.Glu499Val) and the subsequent HA quantitative analysis demonstrated that this mutation resulted in a decreased catalytic ability of HAS2 evidently. As far as we know, the current study is the first to provide important evidence that HAS2 is involved in the etiology of non-syndromic VSD in Chinese population and the mutation of HAS2 plays a potential causative role in the progress of VSD.
